# Understanding the Basis of Drug Resistance of the Mutants of αβ-Tubulin Dimer *via* Molecular Dynamics Simulations

**DOI:** 10.1371/journal.pone.0042351

**Published:** 2012-08-07

**Authors:** Kathiresan Natarajan, Sanjib Senapati

**Affiliations:** Department of Biotechnology, Indian Institute of Technology Madras, Chennai, India; Complutense University, Spain

## Abstract

The vital role of tubulin dimer in cell division makes it an attractive drug target. Drugs that target tubulin showed significant clinical success in treating various cancers. However, the efficacy of these drugs is attenuated by the emergence of tubulin mutants that are unsusceptible to several classes of tubulin binding drugs. The molecular basis of drug resistance of the tubulin mutants is yet to be unraveled. Here, we employ molecular dynamics simulations, protein-ligand docking, and MMPB(GB)SA analyses to examine the binding of anticancer drugs, taxol and epothilone to the reported point mutants of tubulin - T274I, R282Q, and Q292E. Results suggest that the mutations significantly alter the tubulin structure and dynamics, thereby weaken the interactions and binding of the drugs, primarily by modifying the M loop conformation and enlarging the pocket volume. Interestingly, these mutations also affect the tubulin distal sites that are associated with microtubule building processes.

## Introduction

Microtubules (MT) are dynamic cytoskeletal polymers made up of αβ-tubulin heterodimers. They are involved in various critical cellular events such as cell division, cell motility, maintenance of cell shape, cell polarity, intracellular transport, activity of cell surface receptors etc [1]–[3]. Hence, the tubulins and MTs have become important and attractive drug targets for cancer therapy. Various microtubule stabilizing drugs such as taxanes and epothilones and destabilizing drugs such as colchicines and vinca alkaloids, bind to various sites in tubulin dimer and modulate the MT building processes [4]–[6].

**Figure 1:**
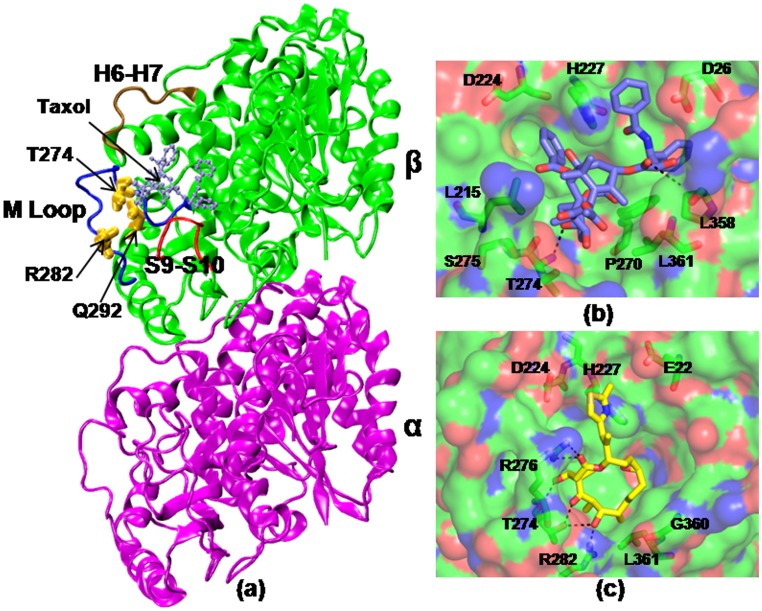
Tubulin-drug interactions. (a) Crystal structure of taxol bound αβ-tubulin dimer (1JFF). The mutated residues are highlighted in yellow and the taxol/epothilone (ice blue) binding site is noted. A few functionally important loops, such as M, H6–H7, S9–S10 are labeled. The protein residues that involve in direct interactions with the drugs are shown in (b) and (c). Taxol (violet) and epothilone (yellow) are shown in licorice representations.

Among microtubule stabilizing drugs, taxanes are widely used in the treatment of lung, breast, ovarian, prostrate cancers and AIDS related Kaposi’s sarcoma [7]. Epothilones are another class of microtubule stabilizing drugs, used in the treatment of advanced breast cancer and in a subset of paclitaxel refractory tumors [8]. Although these 16 membered macrolides are structurally dissimilar to taxol, they show microtubule stabilizing mechanism similar to taxol [9]. Both the drugs bind to a common binding pocket in the intermediate domain of β-tubulin (Figure 1) [10]–[12]. Crystallographic studies, however, have shown that the binding motifs of these two drugs are quite different and unique [13], [14]. Both taxol and epothilone still continue to serve as the lead compounds for the development of new antimitotic drugs. Although these anti-mitotic drugs have been successful in treating various cancers, their efficacy is severely limited by the emergence of tubulin mutants, which are unsusceptible to several classes of tubulin inhibitors.

Cancer cells could acquire drug resistance through multiple factors. The major mechanisms reported to be involved in antimicrotubule drug resistance include: alterations in tubulin dimer due to mutations, alteration in microtubule dynamics, alteration in tubulin isotype expression, and modifications in microtubule regulatory proteins [6]. A large number of recent studies have noted tubulin mutation as a key player in drug resistance [15]–[24]. These studies have particularly pointed the involvement of β-tubulin in drug resistance [18]–[24]. Nonetheless, the detailed mechanism of drug resistance due to β-tubulin mutation is yet to be clearly understood. Efforts dedicated to understand the mechanism of drug resistance are likely to harvest the long term benefits in future drug designing approaches.

Our current effort is to understand how drug resistance could arise due to β-tubulin mutations. Particularly, we are interested in examining the effect of three β-tubulin point mutations, T274I [20]–[22], R282Q [21], [22], and Q292E [22]–[24] that are reported to show cross resistance phenotype to both taxol and epothilone (Table 1). These residues reside in the vicinity of taxol/epothilone binding pocket and undergo mutation upon exposure to certain drugs [21]–[23]. T274 is situated at one end of the M-loop in taxol/epothilone binding pocket and interacts with ether oxygen of Taxol’s octane ring or with C3, C5, and C7 triad of oxygen atoms of epothilone. R282 is situated in the amino terminus of the M-Loop and has direct interaction with the taxane ring in the bound conformation. In bound form with epothilone, it is hydrogen bonded to 7-OH of epothilone. Q292 is not in direct contact with bound taxol or epothilone molecule, rather situated in helix H9 that plays critical role in inter-dimer interactions [24]. In crystal structures, this residue is seen to be ∼7.5 Å away from the octane ring of taxol or ketone oxygen of epothilone and lies opposite to the M-loop.

**Table 1:** Resistant β-tubulin mutations selected for computational study.

Mutation	Cell Type	Phenotype	Reference
T274I	Human	Decreased MT stabilization, epothilone resistant, reduced sensitivity to taxol	20–22
R282Q	Human	Decreased MT stabilization, epothilone resistant, reduced sensitivity to taxol	21,22
Q292E	Human	Decreased MT stabilization, epothilone resistant, reduced sensitivity to taxol	22–24

The above mentioned mutations are introduced to tubulin dimer *in silico* and the effects are investigated *via* all-atom molecular dynamics simulations, protein-ligand docking, and MMPB(GB)SA analyses. Improved insights of structural and dynamic properties of tubulin mutants will be helpful in future drug designing approaches and in ameliorating the efficacy of these drugs. Although many experimental studies have been reported in recent past on β-tubulin mutations, to our knowledge this is the first molecular dynamics study to unravel the detailed mechanism of drug resistance of β-tubulin mutations.

**Figure 2:**
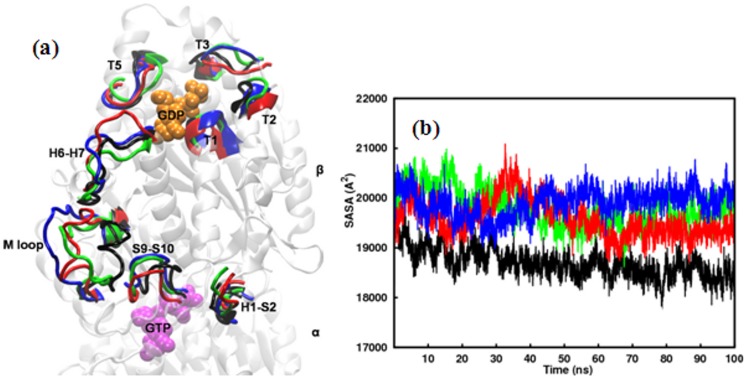
Structural Changes in tubulin mutants relative to WT. a) Time-averaged structures of the β-subunit of tubulin mutants superposed on the time-averaged structure of the β-subunit of WT tubulin. Secondary structural elements which underwent the most significant conformational changes are highlighted. b) Solvent accessible surface area of the β-subunit of WT tubulin and the mutants as a function of time. Color scheme: Wild-type (black), T274I mutation (blue), R282Q mutation (red), Q292E mutation (green).

## Materials and Methods

### Molecular Dynamic Simulations of Wild-type and Mutants

The atomic coordinates for wild-type (WT) tubulin dimer was obtained by engineering the crystal structure of taxol-bound tubulin, downloaded from protein data bank (PDB ID: 1JFF [13]). The crystal structure contained 1 taxol, 422 amino acid residues, 1 GTP, and 1 Mg^2+^ in α-tubulin; 426 amino acid residues, and 1 GDP in β-tubulin. The taxol was removed from the complex. The coordinates for missing residues α:1, β:1, and α:35–60 were modeled using the InsightII graphics package [25]. The hydrogens for heavy atoms were added by leap program in Amber 11.0 package [26]. Added hydrogens were energy minimized for 2000 steps using the steepest descent algorithm. The protonation states of histidines - HID or HIE - were determined by the local hydrogen bonding network using WHATIF program [27]. A set of partial atomic charges for GDP and GTP was obtained *via* quantum electronic structure calculations. Using the Gaussian 09 program [28] with the 6–31+G* basis set, we performed a Hartree-Fock geometry optimization procedure. The atom-centered RESP charges [29] were determined *via* fits to the electrostatic potentials obtained from the calculated wave functions. The missing interaction parameters in the nucleotides were generated using antechamber tools in Amber.

**Figure 3:**
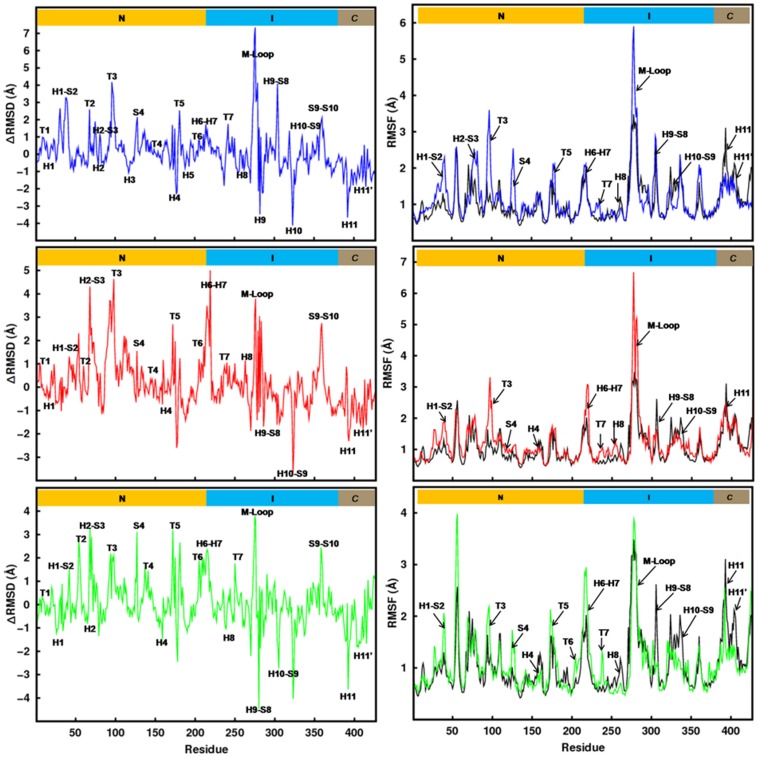
Residue-level displacements and fluctuations. Difference in average C_α_-RMSD of β-tubulin residues of tubulin mutants and the WT (left column) and comparison of average C_α_-RMSF of β-tubulin residues of mutants and the WT (right column). Color scheme is similar to Fig. 2.

After relaxing the added atoms in gas phase, the structure was solvated in an octahedral periodic box of explicit water with water molecules extending 12 Å outside the protein on all sides. The 3-site TIP3P model was chosen to describe the water molecules. To neutralize the system and to maintain an ionic strength of 140 mM, 125 potassium and 89 chloride ions were incorporated. Particle-Mesh Ewald sum [30] with a 10 Å cutoff was used to treat the long-range electrostatics. SHAKE was used to constrain bond lengths between heavy atoms and hydrogens. Noting that the crystal structure used to initiate the MD simulations was determined at low resolution (e.g. in 1JFF, 15% of the rotamer and 10% of the backbone torsion angles were flagged as outliers), an extensive set of minimization and thermalization of the engineered structure was performed to allow the system to remediate the bad geometry and to relax from its lattice-constrained conformation. The system was then equilibrated for 20 ns in NPT ensemble, with a simulation time step of 2 fs. During this period, the energy components, mass density, and root-mean-square-deviations were seen to be converging. The resulting structure, thus, produces us a reliable starting model for the wild-type tubulin. This structure was (i) further simulated to generate the 100 ns production data for wild-type tubulin, and (ii) further engineered to prepare the mutant tubulins, as the following. In the equilibrated structure of wild-type tubulin, respective point mutations were introduced. The resultant systems were further equilibrated for 20 ns following the same procedure as described above. Subsequently, a production phase of 100 ns was generated for each mutant system. All simulations were performed using the NAMD package [31] with AMBERFF99SB force field [32] on 64 processors of an Infiniband Xeon E5472 linux cluster. The data was saved at an interval of 2 ps for analyses.

**Figure 4:**
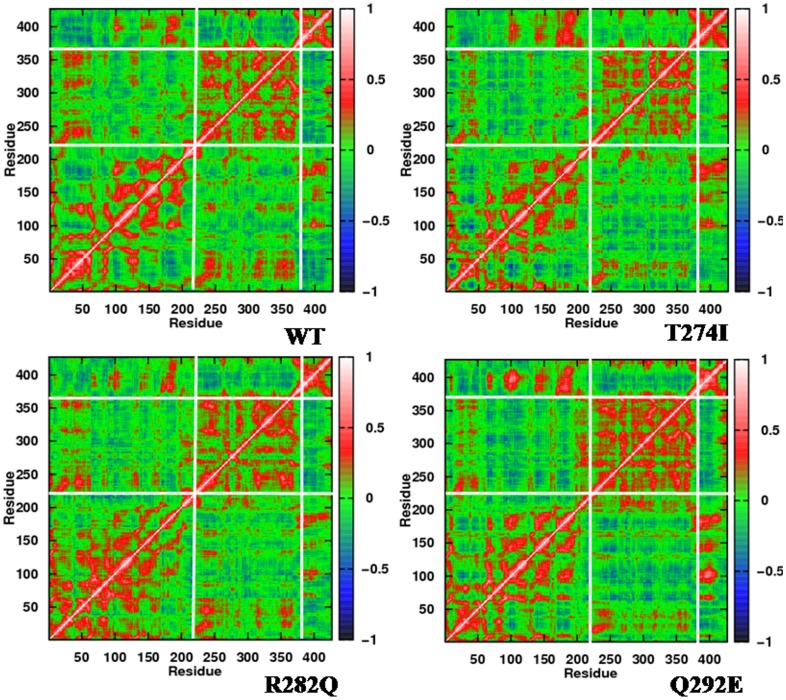
Correlations of the motions of various regions in β-tubulin. Two dimensional cross-correlation maps of the β-subunit of WT and mutated tubulins. Red patches indicate the positively correlated motions, whereas blue patches indicate anti-correlated motion. The maps have been calculated for the C_α_ aoms from the final 10 ns MD data. Very similar patterns were obtained when the maps were generated on other sets of 10 ns data.

Simulation trajectories were used to compute various properties of the protein, including the correlation of motions among its residues in various regions. The correlation analysis was obtained by examining the dynamic cross correlation map (DCCM) of the Cα atoms. The matrix element C_ij_ in DCCM reads as:


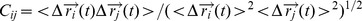


where <> denotes an MD-averaged quantity and 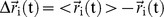 the displacement from the average MD position 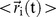 of atom i during a generic MD step. C_ij_ varies from −1.0 for completely anticorrelated motions to +1 for completely correlated motions. A value close to +1 reflects a high correlation between the motions of a pair of Cα atoms. The largest values are obviously found for Cα atoms belonging to residues i and i±a with a = 0, 1, 2 (diagonal elements in the map).

### Protein-ligand Docking

To advance our knowledge on mutant-drug interactions, we performed protein-ligand docking studies through autodock 4.2 [33]. Autodock searches ligand conformations comprehensively and estimates the free energy of its binding to the target. It uses amber force field and a free energy scoring function based on linear regression analysis [34]. As controls, first taxol and epothilone A were docked on the equilibrated structures of WT tubulin. Subsequently, the drugs were docked on the mutants. Gasteiger charges were assigned to all atoms and rotatable bonds were assigned using AutoDockTools. The binding energies between protein and ligands were calculated using atom affinity potentials pre-calculated on grid maps using AutoGrid. The affinity grid maps centered on the active site had dimensions of 80×80×80 Å with 0.375 Å spacing between grid points. Autodock 4.2 estimates the ligand binding through the conformational search space using Lamarckian genetic algorithm. Three hundred trails were performed for wild-type and each mutant. The final docked conformations were clustered using a tolerance of 1 Å root-mean-square deviation (RMSD).

**Figure 5:**
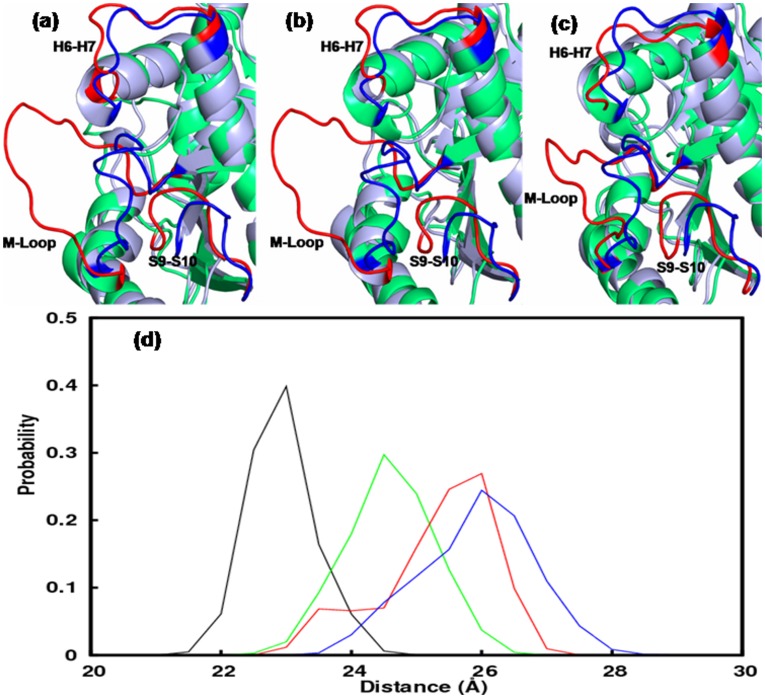
Local changes due to mutations in β-tubulin. The conformations of the drug binding loops, M, H6–H7, S9–S10, in mutants and the WT: a) T274I mutation, b) R282Q mutation and c) Q292E mutation. The loops in mutants are colored red and those in the WT are colored blue. d) Probability distribution of the M loop conformations in mutants and WT. Color scheme is similar to Fig. 2.

### MMPBSA and MMGBSA Analyses

In order to get better understanding of ligand binding, the lowest energy docked complex from each class was further simulated for 10 ns in explicit water. The binding energies of the drugs were computed from the simulation trajectories, using molecular mechanics Poisson–Boltzmann surface area (MMPBSA) and molecular mechanics generalized Born surface area (MMGBSA) approaches [35], [36]. For this purpose, a total of four windows with 50 snapshots at 10 ps intervals in each (i.e. last 2 ns data) were taken from the trajectory and the interaction energies were calculated using the scripts provided in the AMBER 11 package [26]. The binding free energy of the receptor-ligand binding (ΔG_bind_) is calculated by taking the difference between the free energies of the receptor-ligand complex (G_complex_) and the unbound receptor (G_receptor_) and ligand (G_ligand_):


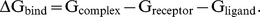


The ΔG_bind_ is composed of the changes in the molecular mechanical gas phase energy (ΔE_MM_), entropic contribution, and solvation free energy:


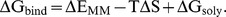


ΔG_solv_ is estimated by either solving the linearised Poisson Boltzman or Generalized Born equation for each of the three states (ΔG_polar_) and adding an empirical term for hydrophobic contributions to it (ΔG_nonpolar_). The hydrophobic contribution is calculated from the solvent accessible surface area. The entropic contribution is omitted from the calculation for simplicity [37], [38].

**Table 2:** Binding energetics of taxol in wild type and mutated tubulins from docking studies.

SYSTEM	ΔG_binding_ (kcal/mol)	K_I_ (µM)	RMSD_Ligand_ from docking(Å)	RMSD_Ligand_ after simulation(Å)	RMSD_Protein_ from docking(Å)	RMSD_Protein_ after simulation(Å)
**WT**	−7.8	2.08	1.57	1.32	1.93	1.74
**T274I**	−5.29	140.54	6.92	5.48	2.35	2.73
**R282Q**	−4.69	383.18	6.19	4.25	2.52	3.04
**Q292E**	−5.49	100.05	5.48	2.81	2.18	2.68

Binding energies are obtained from the lowest energy taxol-tubulin docked complexes. For WT tubulin, the experimental K_I_ = 2.5 µM [42]. Also listed are the RMSD values of the protein and ligand, relative to the crystal structure.

**Table 3:** Binding energetics of epothilone A in wild-type and mutated tubulins from docking studies.

SYSTEM	ΔG_binding_ (kcal/mol)	K_I_ (µM)	RMSD_Ligand_ from docking (Å)	RMSD_Ligand_ after simulation (Å)	RMSD_Protein_ from docking(Å)	RMSD_Protein_ after simulation (Å)
**WT**	−7.79	2.11	1.77	1.40	1.96	1.85
**T274I**	−6.71	12.92	6.31	8.37	2.33	2.87
**R282Q**	−5.96	45.48	6.28	3.27	2.58	2.96
**Q292E**	−6.82	10.75	3.22	2.46	2.13	2.62

Binding energies are obtained from the lowest energy epothilone-tubulin docked complexes. For WT tubulin, the experimental K_I_ = 1.4 µM [43]. Also listed are the RMSD values of the protein and ligand, relative to the crystal structure.

**Table 4:** Binding energetics of taxol in wild type and mutated tubulins using the MMPBSA and MMGBSA methods.

SYSTEM	ΔG_binding; PB_ (kcal/mol)	K_I, PB_ (µM)	ΔG_binding, GB_ (kcal/mol)	K_I, GB_ (µM)
**WT**	−7.70±0.18	2.47	−7.65±0.12	2.67
**T274I**	−4.48±0.97	547.58	−4.35±0.36	682.32
**R282Q**	−5.81±0.89	58.53	−5.31±0.65	135.44
**Q292E**	−6.42±0.25	21.02	−6.23±0.21	28.92

The error bars calculated from four separate windows are included.

**Table 5:** Binding energetics of epothilone A in wild type and mutated tubulins using the MMPBSA and MMGBSA methods.

SYSTEM	ΔG_binding, PB_ (kcal/mol)	K_I, PB_ (µM)	ΔG_binding, GB_ (kcal/mol)	K_I, GB_ (µM)
**WT**	−7.89±0.27	1.79	−7.82±0.39	2.01
**T274I**	−4.89±0.75	274.65	−4.63±0.52	423.48
**R282Q**	−6.26±0.63	27.51	−5.98±0.46	44.01
**Q292E**	−6.65±0.42	14.38	−6.45±0.35	19.96

The error bars calculated from four separate windows are included.

**Figure 6:**
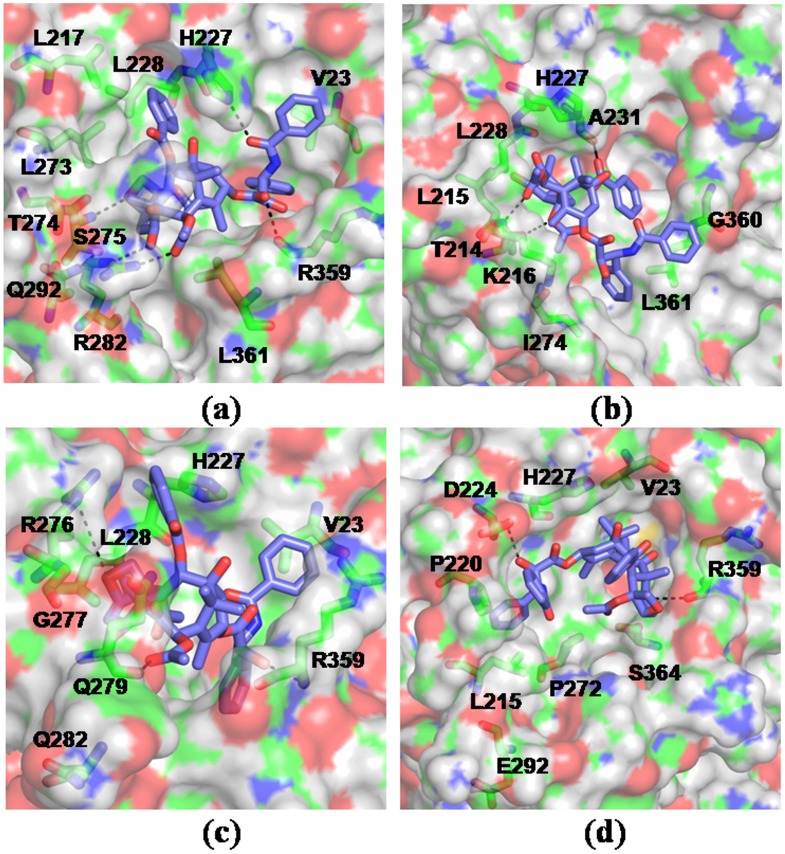
Binding motif of taxol in WT and mutated tubulins. The drug-receptor complexes were obtained by simulating the lowest energy docked complexes for 10 ns in explicit water. Taxol is shown in licorice and the tubulin residues involved in interactions are colored according to atom type – green: C, red: O, blue: N, white: H. Results shown for - a) WT b) T274I c) R282Q d) Q292E. Mutations resulted in altered mode of drug binding and loss of characteristic drug-receptor contacts.

## Results and Discussion

To examine the effects of reported β-tubulin point mutations on tubulin structure and dynamics, we performed all atom molecular dynamics simulations of WT and three tubulin mutants. Each simulation was carried out for at least 100 ns in production phase.

### Mutations Affect the Structure and Dynamics of Tubulin Dimer

The comparison of trajectories from wild-type and mutant simulations implies that both WT tubulin and mutants undergo structural changes and deviate significantly from the taxol bound crystal structure. Also, the structures of the mutants differ from the WT. This can be seen from Fig. S1, where we have plotted the distribution of root mean squared displacements (RMSD) of the protein. RMSD values were calculated for each frame along the trajectory with respect to the crystal structure. The distributions clearly indicate that the extent of changes experienced by WT and the mutants is different. In Gaussian-like distributions, majority of the mutant frames exhibit larger RMSD than the WT tubulin. Among the two subunits of tubulin dimer, β-tubulin has undergone relatively larger conformational changes than α-tubulin (data shown latter). Fig. S2 shows the time evolution of radius of gyration (Rg) of β-tubulin in WT and mutants. Rg values essentially provide an insight about the size of the protein. The stable Rg values along the trajectories indicate that the overall packing of β-tubulin is maintained during simulations. The slightly larger Rg values for the mutants imply a possible expansion of β-tubulin structure due to mutations.

**Figure 7:**
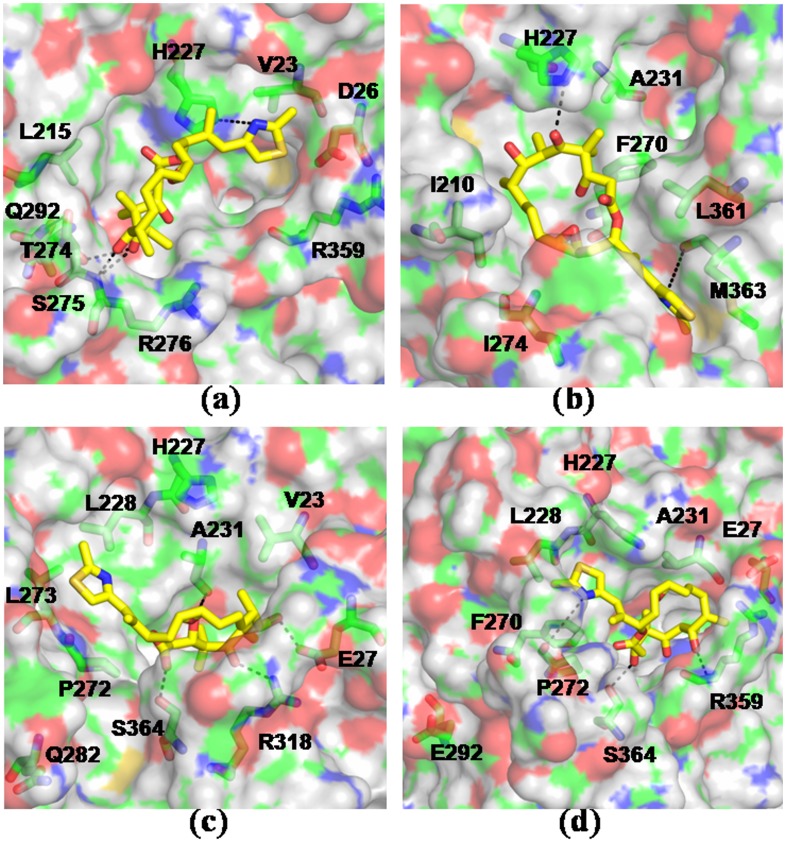
Binding motif of epothilone A in WT and mutated tubulins. The drug-receptor complexes were obtained by simulating the lowest energy docked complexes for 10 ns in explicit water. Results shown for - a) WT b) T274I c) R282Q d) Q292E. Epothilone A is shown in yellow.

A detailed structural comparison between the β-subunits of WT and mutants is presented in Fig. 2a. The figure is generated by a stereo superposition of the average structures of WT and mutants from final 10 ns simulations, according to C_α_ atoms of the β-subunits. In a 3D representation of the structural elements, the figure highlights the most notable variances in WT and mutants. Larger fluctuations are observed in the functionally relevant loops of mutants compared to the WT. The sites of maximum variances include M loop, H6–H7 loop, and S9–S10 loop in the taxol-binding I-domain and nucleotide-binding loops T1, T2, T3, T5 and H1–S2 in the N-domain. The T3 and T5 loops, which are crucial for binding the nucleotide and for longitudinal contacts in the protofilament show larger deviations in mutants. During simulations, the M loop that comprises part of the taxol or epothilone binding site and involves in lateral interactions along the protofilament exhibited a great deal of variation and fluctuates greatly in mutants. While it remains in a stable inward conformation in WT, it protrudes outward laterally in all mutants. The H1–S2 loop that resides opposite to M loop also demonstrates a greater outward movement in mutants. The H6–H7 loop, which is known to play a key role in longitudinal interactions along the protofilaments, also shows greater extendibility in mutants than the WT. These changes are shown in Fig. 2a.

**Table 6:** Calculated mean values for various properties, their standard deviations, and the differences between the mean values of wild type and mutated tubulins.

Radius of gyration of β-tubulin (Å)			
SYSTEM	MEAN	Standard deviation	ΔRg (Mut-WT)
**WT**	21.17	0.05	–
**T274I**	21.37	0.09	0.20
**R282Q**	21.29	0.06	0.12
**Q292E**	21.40	0.08	0.23
**SASA of β-tubulin (Å^2^)**			
**WT**	18696.28	293.26	–
**T274I**	19906.59	292.78	1210.31
**R282Q**	19581.86	371.66	885.58
**Q292E**	19813.09	367.25	1116.81
**Hydrophilic SASA of β-tubulin (Å^2^)**			
**WT**	14085.81	236.11	–
**T274I**	14723.03	237.69	637.22
**R282Q**	14764.84	228.77	679.03
**Q292E**	14778.91	246.82	693.10
**Hydrophobic SASA of β-tubulin (Å^2^)**			
**WT**	4610.47	112.97	–
**T274I**	5183.56	147.39	573.09
**R282Q**	4817.02	218.27	206.55
**Q292E**	5034.18	176.94	423.71
**M loop Distance (Å)**			
**WT**	22.68	0.49	–
**T274I**	25.67	0.87	2.99
**R282Q**	25.08	0.87	2.40
**Q292E**	24.34	0.67	1.66

It will be interesting to see whether the effect of mutations is also reflected on the solvent accessible surface area (SASA). For that, we have calculated SASA for β-tubulin of WT and mutants by rolling a spherical probe of radius 1.4 Å across the protein surface. Time dependence of total SASA, as shown in Fig. 2b, shows that the mutants experience more solvent exposure than the WT. This observation is in accordance with the increased Rg values of the mutants. A similar trend was also observed when total SASA was decomposed into hydrophobic and hydrophilic SASA for the constituent residues in β-tubulin (Fig. S3).

### Internal Motions in β-tubulin are More Sensitive to Mutations

To understand the effect of these point mutations further, we calculated RMSD differences between the WT and the mutants (ΔRMSD  =  RMSD_mutant_ – RMSD_WT_). The left column graphs in Fig. 3 show the ΔRMSD for all β-tubulin residues in the three mutants. The ΔRMSD values indicate that the mutations significantly affect the secondary structural elements of both I-domain and N-domain. The increased ΔRMSD around the mutated site suggests that mutations perturb taxol/epothilone binding pocket significantly. Moreover, the observed high ΔRMSD values in the critical regions of N-domain suggest that, introduced mutations in I-domain allosterically influence the N-domain of tubulin dimer. The corresponding ΔRMSD values for α-tubulin (Fig. S4a) suggest that most of the conformational changes due to mutations are localized in β-tubulin.

To examine the local structural transformations of β-tubulin in greater detail, the RMSF of each residue was calculated. RMSF essentially calculates the degree of movement of each Cα around its average position, implying regions of the protein that are highly flexible will show a large RMSF value while regions that are constrained will show up a low RMSF. The right column graphs in Fig. 3 compare the relative fluctuations of β-tubulin residues in WT and mutants. It is clear that the mutants adopt very different dynamic behavior compared to the wild-type tubulin. The fluctuations in the mutants are higher than the WT almost in all residues. Comparison further indicates that the regions of high flexibility include regions close to the mutated sites and some distal sites in N-domain. The M-loop in T274I and R282Q mutants is very flexible compared to WT. The adjacent regions, *e.g.* H6–H7, T7 also experience an increased flexibility. In Q292E mutant, the mutated residue moves to a solvent exposed conformation and forms a saltbridge with L297 stabilizing the M loop in an open conformation and making it less flexible compared to other two mutants. The distal sites that undergone most significant changes upon mutations are the various nucleotide binding loops, T3, T5, T7 etc. These loops not only involve in nucleotide binding, but are also known to involve in the dimer-dimer longitudinal interactions along protofilament. The increased flexibility observed in these loops is, therefore, likely to have significant influence on protofilament stability and dynamics. The mutations introduced in the I-domain influencing the N-domain again suggest the possibility of allosteric coupling between I-domain and N-domain. The result is consistent with the finding of Mitra and Sept [39], where the authors have reported an allosteric communication between I-domain and N-doamin due to taxol binding. It is interesting to see here that the point mutations introduced in the taxol binding pocket also show similar allosteric changes in the nucleotide binding domain. The corresponding RMSF values for α-tubulin show smaller changes (Fig. S4b).

Correlations of the motion among various regions in β-subunit can be obtained by examining the dynamic cross correlation map (DCCM) of the Cα atoms. Fig. 4 shows the DCCM of WT tubulin and its three mutants. Mutants show a general increase in residue-residue correlations, both in the I-domain and N-domain (denser red) compared to WT. Moreover, the mutations are seen to affect the correlation of residues that are sequentially and spatially apart. The anti-correlationship, as present between N- and I- domain in WT, increases to some degree in the mutants (red patches around residue 250–300 become weaker and blue patches become denser). This again signifies an allosteric communication between N- and I-domain, wherein the introduced mutations in I-domain induce changes in N-domain. The increased residue-residue correlations indicates that the flexibility arising due to the mutations around the mutated sites are not decoupled from other motions in the structure.

### Mutations Perturb Taxol/epothililone Binding Pocket

The local changes due to mutations are found to be even more prominent. All the mutants perturbed the taxol/epothilone binding region quite significantly. The most notable change was observed for M-loop. Fig. 5 shows a comparison of the taxol/epothilone binding pocket by superposing the time-averaged structures of WT and mutants. M-loop is observed to attain distinctly different conformation in all mutants, where it is completely pushed out of the groove (Figs. 5a–c). This can reduce the compactness of the tubulin structure significantly, as was also observed from larger Rg and SASA values. This implies that the residues T274, R282, and Q292 are critical for maintaining the proper M loop conformation. Recall that M-loop is an important structural motif that involves in lateral interactions to confer stability to MT, apart from being involved in drug binding. Fig. 5d shows probability distribution of the M loop movement in β-tubulin of WT and mutants. It is clear that M-loop becomes less compact and more flexible in the mutants. The distribution was obtained by calculating the instantaneous distances between the centre of masses of M loop (residues 270–286) and whole β-tubulin. In wild-type, M loop distribution was nearly Gaussian, peaking at an average distance of 23 Å. On the other hand, mutants’ peak observed at an average distance of 25 to 27 Å, implying a wide open state of M-loop. We rank the degree of opening of M-loop in mutants and WT as, T274I> R282Q>Q292E>WT. Significant local changes were also observed for loops H6–H7 and S9–S10, which also surround the taxol binding pocket (Fig. 5a–c). It will be interesting to quantify the average volume of the taxol/epothilone binding pocket in these mutants. The average volumes were calculated to be: WT = 498 Å^3^, T274I = 1112 Å^3^, R282Q = 804 Å^3^, and Q292E = 750 Å^3^. The pocket volume was calculated through LIGSITE algorithm, where a probe was allowed to scan seven individual directions (four cubic diagonals and x, y, and z directions) along a protein structure projected onto a 3D grid, to calculate the number of solvent-surface-solvent events [40]. The increased volume of the binding pocket in mutants could contribute to drug resistance by weakening the drug-receptor interactions.

### Mutations Result in Reduced Binding of Taxol and Epothilone

To compare the drug-receptor interactions, molecular docking studies were carried out for taxol and epothilone A with the simulation-generated model structures of WT and mutated tubulins. Three hundred trails were performed for wild-type and each mutant. Results indicate that the mutations render weaker interaction with the drugs, primarily due to the loss of favorable interactions of the drugs with the M-loop and adjacent residues. The calculated values of free energy of binding of the drugs to the receptor, in the lowest energy complexes, are presented in Tables 2 and 3. As the tables indicate, mutants have much weaker binding with taxol and epothilone than WT. Detailed analysis show that both taxol and epothilone acquire an altered mode of binding in the mutants. To check if there could be any induced fit in the binding pocket, the lowest energy docked complex from each class was further simulated for 10 ns in explicit water. The systems, particularly the ligand orientations, were seen to experience large changes due to induced fit during the simulations. Tables 2 and 3 also tabulate the changes in terms of the RMSD values of the protein and the drugs. For a better understanding of ligand binding to the tubulin mutants, we also computed the protein-ligand interaction energies from the simulation-generated trajectories *via* MMPBSA and MMGBSA methods. Tables 4 and 5 list the binding free energies of taxol and epothilone from these calculations. The binding of both the drugs was found to be weaker in the mutant variety than WT tubulin irrespective of the method of calculation, suggesting a significant alteration in the binding motifs of the mutants. Figs. 6 and 7 provide a detailed structural representation of the drug-receptor interactions in the simulated complexes.

In WT tubulin, taxol makes polar contacts primarily with residues T274, R282 in the M-loop, H227 in the helix H7, and R359 in the S9–S10 loop; whereas epothilone makes polar contacts with T274, R276 in the M-loop and H227 in the helix H7 (shown in dotted lines). Most of the other ligand-protein contacts were hydrophobic, as was also noted in the crystal structures [13], [14]. In T274I mutant, the mutation disrupts the local intra-protein hydrogen bonds due to the elimination of threonine -OH group. Moreover, the hydrophobicity of the region increases significantly due to the isoleucine residue. As a result, the M-loop swings away from the taxol/epothilone binding pocket, making the lining residues unfavorable for interaction with the drugs (Fig. 6b, 7b). Table S1 and S2 list the interactions present between the drugs and the tubulin mutants in the docked complexes. As the tables show, not only the interaction partner but also the extent of contacts changes due to mutation. The number of polar and hydrophobic contacts in the mutants diminishes sharply in comparison to the WT (see tables S1 and S2). The ligand-protein contacts were calculated based on the interface surface complementarity, and classified by hydrophilic/hydrophobic properties of the contacting ligand and protein atoms [41]. In R282Q mutant, the mutated residue moves to a solvent exposed conformation pushing the M loop to an open state similar to T274I mutation. In such an orientation, the M loop and other interacting residues fail to make favorable polar contacts with taxol or epothilone (Figs. 6c, 7c, Table S1, Table S2). In mutant, taxol makes polar contact primarily with R359 in the S9–S10 loop region, whereas epothilone makes polar contacts with R318 situated in S8 and S364 in S9–S10 loop. The results thus indicate that the positions of residues T274 and R282 are important for favorable drug-tubulin interactions.

In WT tubulin, the residue Q292, residing in helix H9, makes a hydrogen bond with S275 to contribute to M loop stability. The Q → E mutation destabilizes this hydrogen bonding network due to the negative charge on glutamic acid, which induces significant changes in the orientations of M-loop and H6–H7 loop. As a result, the drug binding becomes unfavorable in the pocket. In this mutation, taxol makes polar contacts with D224 situated in the H7 helix and R359 in the S9–S10 loop region (Fig. 6d, Table S1), whereas epothilone makes polar contact with R359 in the S9–S10 loop region (Fig. 7d, Table S2). The results indicate that the polar side chain of glutamine at this position is essential to mediate interactions with other polar or charged residues in the protein and also for effective binding of taxol and epothilone. This observation supports the experimental finding that Q292E mutation exhibits resistance phenotype [22], [23] and fails to undergo drug induced polymerization [24]. Overall, the simulation and docking results suggest that the ineffective binding of taxol and epothilone to tubulin mutants is primarily due to the loss of characteristic protein-drug contacts seen in the crystal structures.

Lastly to check the reliability of the presented results, we compare the noise in the simulation data with the differences between wild type and mutant values. Table 6 includes the mean values of the measured properties, their standard deviations, and the differences between the mean values of those properties in the wild type and mutants. As the table indicates, the differences between the mean values are always substantially larger than the standard deviations. This suggests that the observed changes in tubulin dimers due to mutations are reliable and not the artifacts of calculations.

### Conclusions

We investigated the structural features of wild type αβ-tubulin dimer and its three drug resistant variants through all-atom MD simulations. Results provide atomic-level description of the conformational changes caused by these mutations. Detailed analyses indicate that the mutated tubulins are more flexible than the WT and the mutations can alter the overall structure and dynamics of tubulin dimer. The dominant effect due to mutations was observed in the taxol/epothilone binding pocket. Mutations locally alter the conformation of M-loop, which is crucial for binding drugs, such as taxanes and epothilones. Further, these simulations predict allosteric coupling between nucleotide binding N-domain and taxol/epothilone binding I-domain. Results from protein-ligand docking and MMPB(GB)SA analyses imply that the mutations alter the binding motif of taxol and epothilone significantly and weaken the drug-receptor interactions. Hence, these mutations could affect the microtubule dynamics in two ways. Firstly, it can locally alter the taxol/epothilone binding site in β-tubulin and weakens the drug binding. Secondly, it can modulate the assembly of tubulin dimers *via* allosteric changes in N-domain. Our study provides molecular level explanation for tubulin drug resistance reported in various experimental studies and we expect the insights obtained here would be helpful in future drug designing approaches.

## Supporting Information

Figure S1 The distribution of C_α_ RMSDs of WT tubulin and its mutants. RMSD values were calculated for each frame along the trajectory with respect to the crystal structure.
(TIF)

Figure S2 Time evolution of radius of gyration of the β-subunit of WT tubulin and its mutants. Color scheme: WT (black), T274I mutation (blue), R282Q mutation (red), Q292E mutation (green).
(TIF)

Figure S3 Time evolution of a) hydrophilic SASA b) hydrophobic SASA of the β-subunit of tubulin dimer. Color scheme is similar to Fig. S2.
(TIF)

Figure S4Comparison of a) *Δ* RMSD and b) root mean square fluctuations 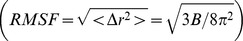 of the C_α_ atoms of α-tubulin in WT and mutants. Color scheme is similar to Fig. S2. The added missing loop region, residues 35–60, shows high fluctuations.(TIF)

Table S1 Interactions between taxol and tubulin in simulated complexes. Nature of interactions and the participating residues are listed. The distance between the closest pair of atoms are noted. The ligand-protein contacts were calculated based on the interface surface complementarity, and classified by hydrophilic/hydrophobic properties of the contacting ligand and protein atoms [41].
(DOC)

Table S2 Interactions between epothilone A and tubulin in simulated complexes. Nature of interactions and the participating residues are listed. The distance between the closest pair of atoms are noted. The ligand-protein contacts were found as similar to Table S1.
(DOC)
