# Supplementary figures and images for: Understanding the Basis of Drug Resistance of the Mutants of αβ-Tubulin Dimer via Molecular Dynamics Simulations
Source: PLoS One. 2012 Aug 7;7(8):e42351. doi: 10.1371/journal.pone.0042351 (PMC3413672; doi:10.1371/journal.pone.0042351)

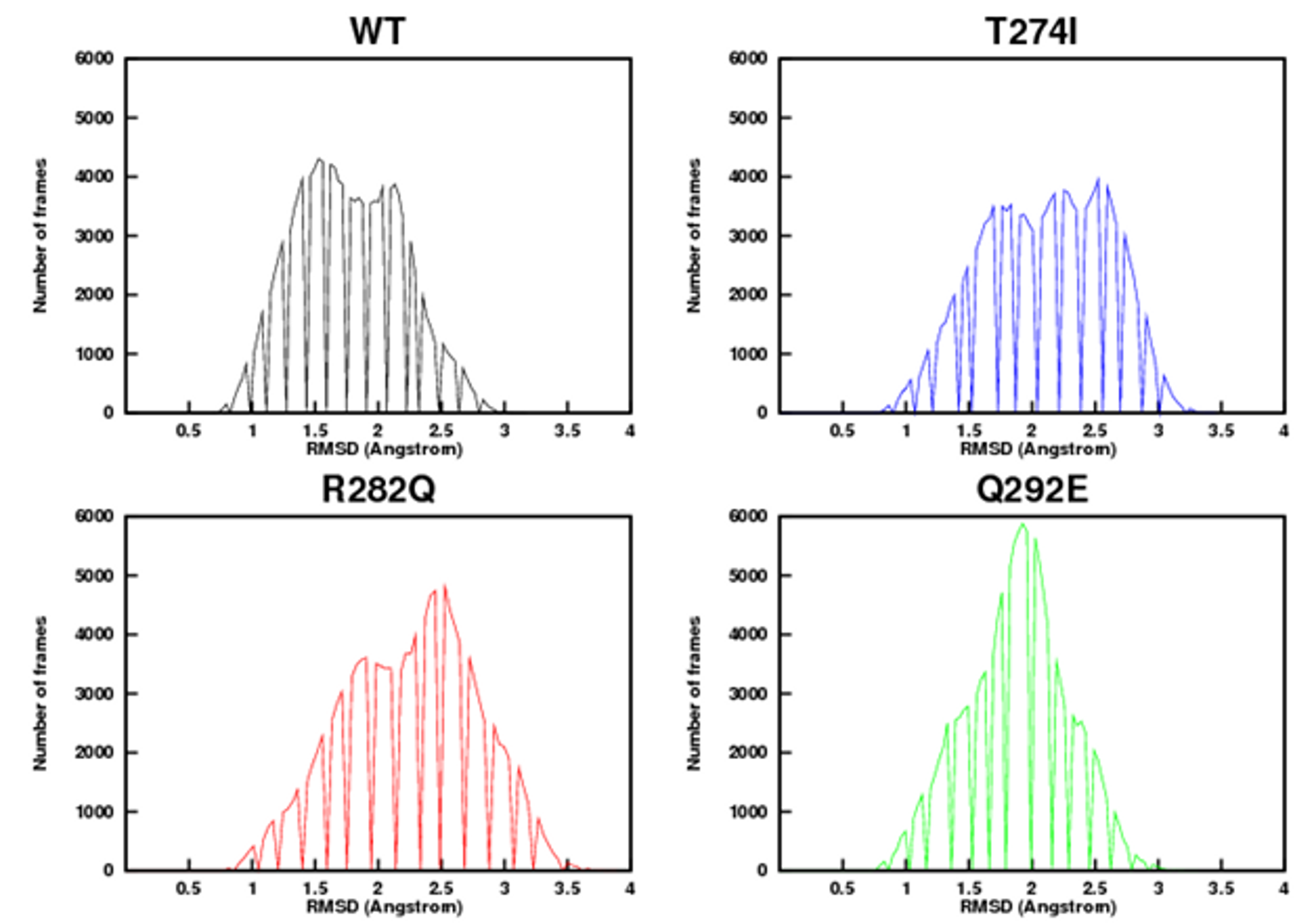

Supplement: Figure S1 — The distribution of Cα RMSDs of WT tubulin and its mutants. RMSD values were calculated for each frame along the trajectory with respect to the crystal structure. (TIF) [file pone.0042351.s001.tif]

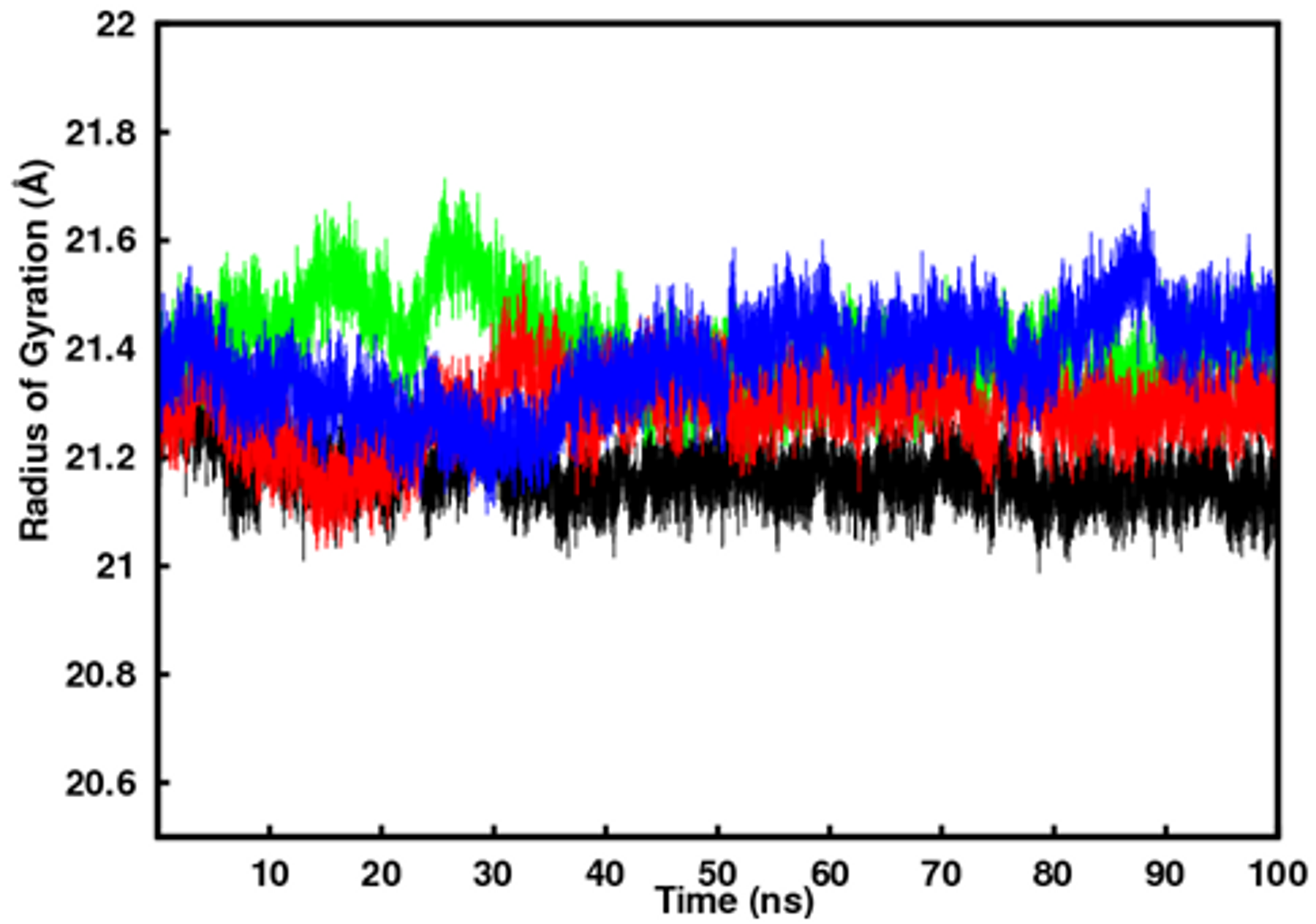

Supplement: Figure S2 — Time evolution of radius of gyration of the β-subunit of WT tubulin and its mutants. Color scheme: WT (black), T274I mutation (blue), R282Q mutation (red), Q292E mutation (green). (TIF) [file pone.0042351.s002.tif]

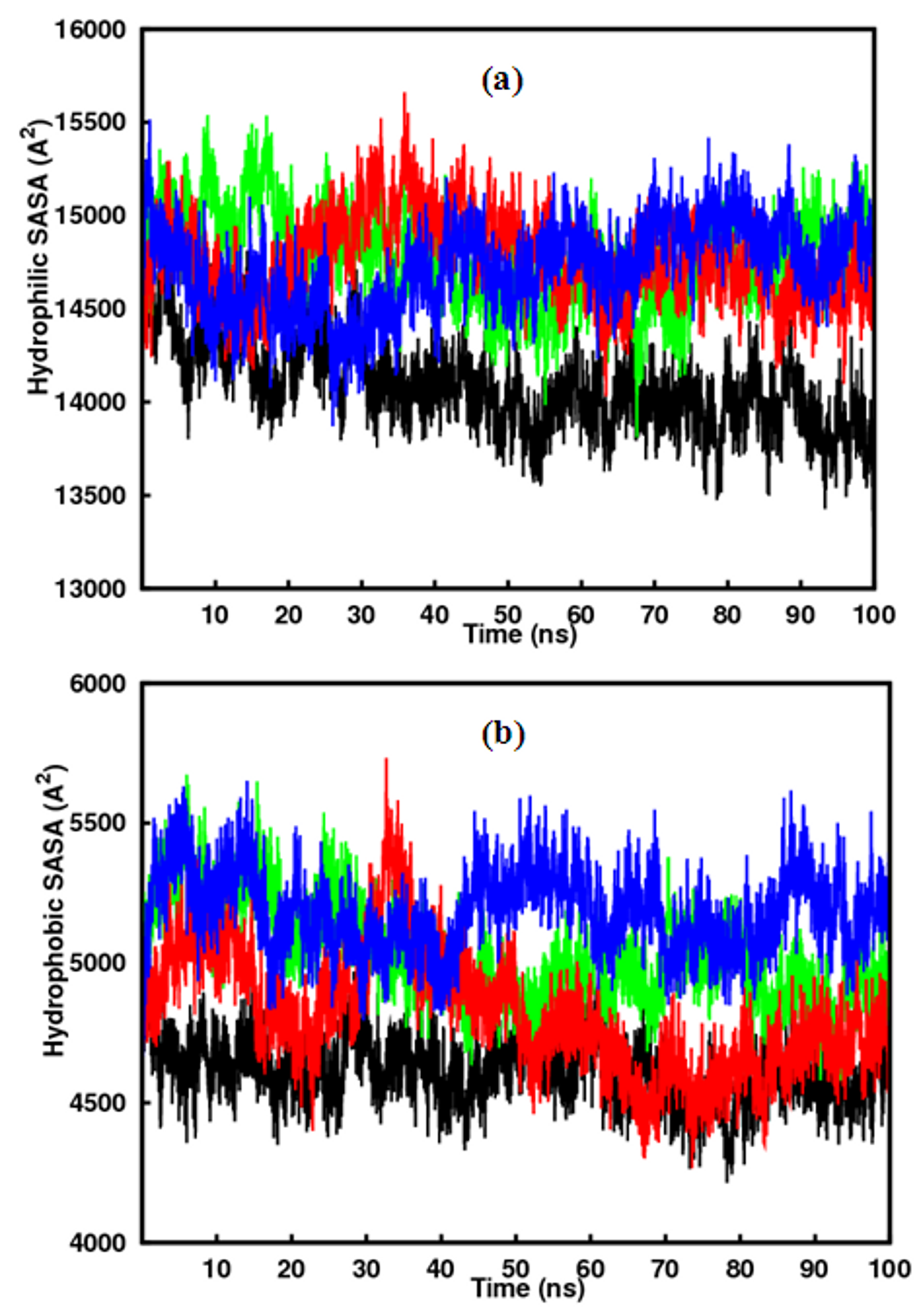

Supplement: Figure S3 — Time evolution of a) hydrophilic SASA b) hydrophobic SASA of the β-subunit of tubulin dimer. Color scheme is similar to Fig. S2. (TIF) [file pone.0042351.s003.tif]

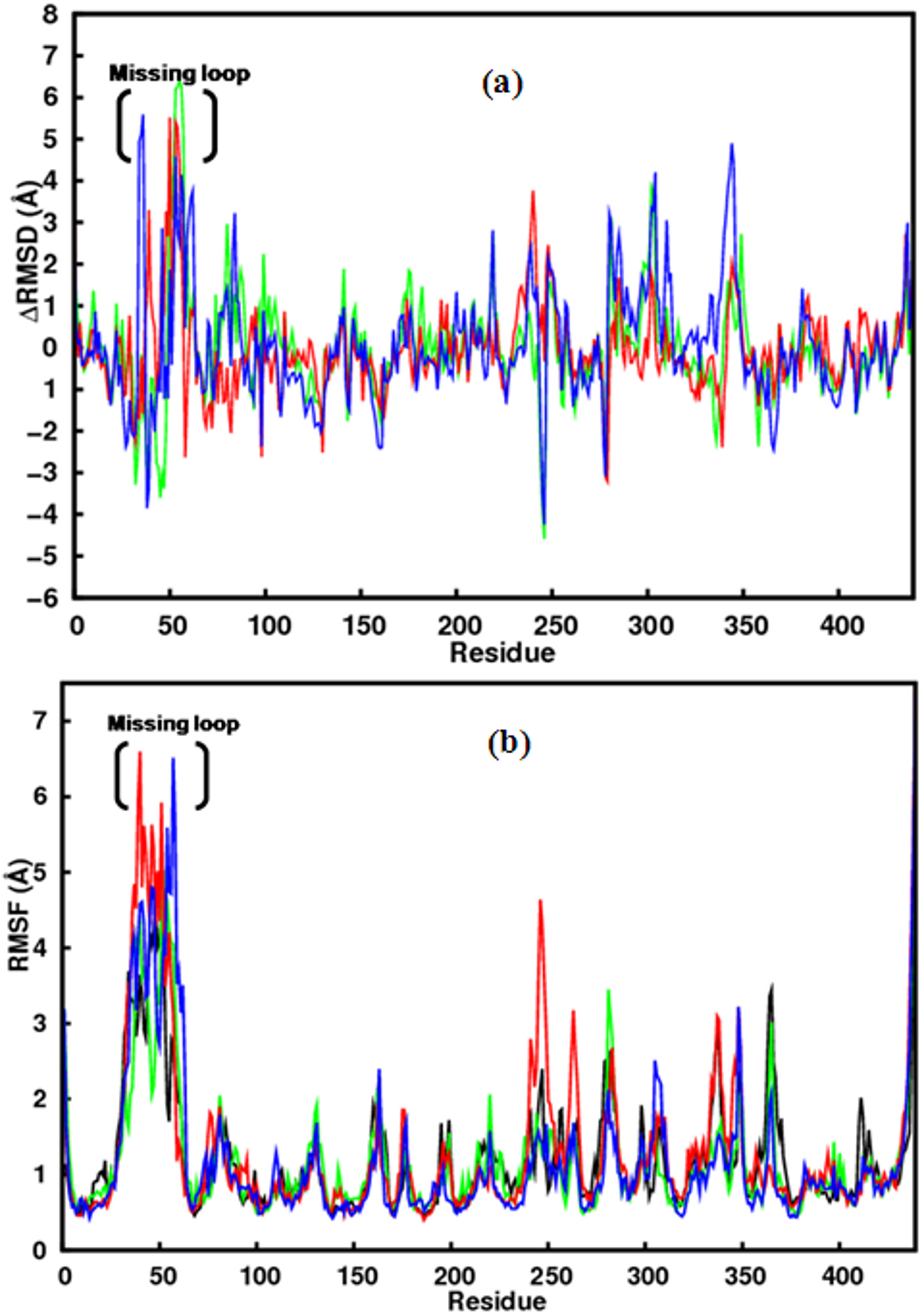

Supplement: Figure S4 — Comparison of a) Δ RMSD and b) root mean square fluctuations of the Cα atoms of α-tubulin in WT and mutants. Color scheme is similar to Fig. S2. The added missing loop region, residues 35–60, shows high fluctuations. (TIF) [file pone.0042351.s004.tif]
