# Supplementary material for: Understanding the Basis of Drug Resistance of the Mutants of αβ-Tubulin Dimer via Molecular Dynamics Simulations
Source: PLoS One. 2012 Aug 7;7(8):e42351. doi: 10.1371/journal.pone.0042351 (PMC3413672; doi:10.1371/journal.pone.0042351)
Supplement: Table S1 — Interactions between taxol and tubulin in simulated complexes. Nature of interactions and the participating residues are listed. The distance between the closest pair of atoms are noted. The ligand-protein contacts were calculated based on the interface surface complementarity, and classified by hydrophilic/hydrophobic properties of the contacting ligand and protein atoms [41]. (DOC) [file pone.0042351.s005.doc]

|  | **Wild-type** | |  | **T274I** | |  | **R282Q** | |  | **Q292E** | |
| --- | --- | --- | --- | --- | --- | --- | --- | --- | --- | --- | --- |
| **Ligand atom** | **Protein**  **Residue** | **Distance**  **(Å)** |  | **Protein**  **Residue** | **Distance**  **(Å)** |  | **Protein**  **Residue** | **Distance (Å)** |  | **Protein**  **Residue** | **Distance**  **(Å)** |
|  |  |  |  |  |  |  |  |  |  |  |  |
| **Hudrogen Bonding/Polar Interaction** | | | | | | | | | | | |
| O3 |  |  |  | A231:3HB | 4.6 |  |  |  |  |  |  |
| O4 |  |  |  | T214:O | 3.4 |  |  |  |  |  |  |
| O6 | T274:N | 2.9 |  |  |  |  | R276:3HG | 2.9 |  |  |  |
| O7 | R282:NE | 3.5 |  |  |  |  | Q279:2HB | 4.4 |  |  |  |
| O8 | R282:NH2 | 4 |  | L215:2HB | 2.7 |  |  |  |  | R359:3HG | 4 |
| O10 | R282:NH2 | 2.8 |  |  |  |  |  |  |  | S364:OG | 5.4 |
| O12 | R359:O | 3.1 |  |  |  |  | R359:2HD | 3.4 |  | L228:N | 4.4 |
| O13 | R282:O | 2.8 |  |  |  |  | R359:2HB | 2.7 |  | D224:O | 2.8 |
| O13 | R282:N | 3.3 |  |  |  |  | R359:N | 3 |  | L229:N | 3.9 |
| O13 | D26:OD2 | 4.1 |  |  |  |  |  |  |  |  |  |
| O14 | H227:NE | 3.1 |  |  |  |  |  |  |  |  |  |
|  |  |  |  |  |  |  |  |  |  |  |  |
| **Hydrophobic Contacts** | | | | | | | | | | | |
| C3 | L215:CD1 | 4.7 |  |  |  |  |  |  |  |  |  |
| C4 | L215:CD1 | 4.2 |  |  |  |  |  |  |  |  |  |
| C5 | L215:CD1 | 3.9 |  | A231:2HB | 4.4 |  |  |  |  |  |  |
| C6 | L215:CD1 | 4 |  | F270:CZ | 3.8 |  |  |  |  |  |  |
| C7 | L228:CD1 | 3.5 |  | L361:3HB | 3.2 |  |  |  |  |  |  |
| C8 | L228:CD1 | 3.9 |  | L361:3HB | 3.1 |  |  |  |  |  |  |
| C9 |  |  |  |  |  |  |  |  |  |  |  |
| C12 | L361:CD1 | 4.2 |  |  |  |  |  |  |  |  |  |
| C13 | L361:CD1 | 3.8 |  | I274:O | 3.7 |  | F270:CE2 | 3.9 |  |  |  |
| C14 | L215:CD2 | 3.7 |  | L273:CD2 | 4.1 |  | R276:CG | 4.4 |  |  |  |
| C15 |  |  |  | L273:CD2 | 3.6 |  |  |  |  | L361:CD2 | 5.4 |
| C16 | T274:OG1 | 3.2 |  | T214:OG1 | 3.6 |  | T274:OG1 | 2.9 |  | R359:O | 3.7 |
| C17 | L361:CD2 | 4.8 |  |  |  |  | L284:CD2 | 5.9 |  | L361:CD1 | 4.4 |
| C19 | R276:CB | 4 |  | L217:CA | 5.2 |  | R276:2HG | 3.6 |  | R359:O | 3.8 |
| C20 |  |  |  | L215:CD1 | 4.3 |  |  |  |  |  |  |
| C22 |  |  |  | K216:3HB | 3.6 |  | L361:CD2 | 5.4 |  |  |  |
| C23 | R276:CZ | 4.1 |  | L217:CD2 | 3.4 |  | Q279:2HG | 3.4 |  | A231:CB | 3.8 |
| C24 |  |  |  |  |  |  | G360:3HA | 3.3 |  |  |  |
| C25 |  |  |  |  |  |  |  |  |  | A231:2HB | 3.2 |
| C26 |  |  |  | K216:3HE | 5.4 |  |  |  |  | A231:2HB | 3.9 |
| C27 |  |  |  | K216:2HE | 5 |  |  |  |  |  |  |
| C28 | P358:CB | 4 |  |  |  |  |  |  |  |  |  |
| C30 | V23:CG2 | 4.9 |  |  |  |  | A231:2HB | 4.5 |  | P220:3HG | 3.4 |
| C31 | V23:CG2 | 4.7 |  |  |  |  |  |  |  | P220:3HG | 3 |
| C32 | V23:CG2 | 4.2 |  |  |  |  |  |  |  | P220:3HG | 3.2 |
| C33 | D26:CB | 4.2 |  | L361:CD1 | 3.1 |  | V23:O | 3.4 |  |  |  |
| C34 | E22:CG | 4.3 |  | L361:CD2 | 3.9 |  | D26:3HB | 3.2 |  | P220:CD | 4.3 |
| C34 | V23:CG2 | 4.3 |  | L361:CD2 | 3.8 |  | V23:CG2 | 3.9 |  |  |  |
| C35 | V23:CG2 | 4.6 |  | I274:CG1 | 4.4 |  | V23:CG2 | 4.3 |  | P220:3HG | 3.3 |
| C36 |  |  |  | L361:CD2 | 3.4 |  |  |  |  |  |  |
| C37 | P358:CB | 4 |  |  |  |  |  |  |  |  |  |
| C38 | P358:CB | 4.4 |  |  |  |  | P272:2HG | 3.3 |  | L228:CD2 | 3.8 |
| C39 | A231:CB | 3.9 |  |  |  |  | P272:2HG | 3.6 |  | L228:CD2 | 3.8 |
| C40 | V23:CG1 | 3.7 |  |  |  |  |  |  |  | C211:3HB | 3.7 |
| C41 | V23:CG1 | 3.2 |  | L361:CD2 | 3.5 |  |  |  |  | P220:2HG | 3 |
| C42 | V23:CG1 | 3.6 |  | L361:CD2 | 3.7 |  |  |  |  | P220:2HG | 3.3 |
| C44 | G360:C | 4.1 |  | K216:2HB | 3 |  | L361:CD1 | 3.8 |  | A231:2HB | 3.3 |
| C46 |  |  |  |  |  |  | G360:2HA | 3.7 |  | A231:2HB | 3.3 |
| C47 | R276:CD | 4.1 |  |  |  |  | Q280:NE2 | 4.6 |  | V23:CG2 | 4.8 |
| O10 |  |  |  | K216:N | 2.8 |  |  |  |  |  |  |
| O13 |  |  |  | R276:2HB | 3.1 |  |  |  |  |  |  |
|  |  |  |  |  |  |  |  |  |  |  |  |
| **Aromatic Contacts** | | | | | | | | | | | |
| C38 |  |  |  |  |  |  | F270:CZ | 3.6 |  |  |  |
| C39 | F270:CZ | 4.2 |  |  |  |  | F270:CE1 | 3.6 |  |  |  |
| C40 | F270:CZ | 5 |  |  |  |  | F270:CE1 | 3.9 |  |  |  |
|  |  |  |  |  |  |  |  |  |  |  |  |
