# Supplementary material for: Understanding the Basis of Drug Resistance of the Mutants of αβ-Tubulin Dimer via Molecular Dynamics Simulations
Source: PLoS One. 2012 Aug 7;7(8):e42351. doi: 10.1371/journal.pone.0042351 (PMC3413672; doi:10.1371/journal.pone.0042351)
Supplement: Table S2 — Interactions between epothilone A and tubulin in simulated complexes. Nature of interactions and the participating residues are listed. The distance between the closest pair of atoms are noted. The ligand-protein contacts were found as similar to Table S1. (DOC) [file pone.0042351.s006.doc]

|  | **Wild-type** | |  | **T274I** | |  | **R282Q** | |  | **Q292E** | |
| --- | --- | --- | --- | --- | --- | --- | --- | --- | --- | --- | --- |
| **Ligand atom** | **Protein**  **Residue** | **Distance**  **(Å)** |  | **Protein**  **Residue** | **Distance**  **(Å)** |  | **Protein**  **Residue** | **Distance**  **(Å)** |  | **Protein**  **Residue** | **Distance**  **(Å)** |
|  |  |  |  |  |  |  |  |  |  |  |  |
| **Hydrogen Bonding/Polar Interaction** | | | | | | | | | | | |
| N1 | H227:CD2 | 4.4 |  | M363:2HB | 3.6 |  |  |  |  | P272:O | 4.9 |
| N1 | E22:OE1 | 4.9 |  |  |  |  |  |  |  |  |  |
| O2 | H227:ND1 | 3.9 |  |  |  |  | S364:OG | 2.7 |  |  |  |
| O4 | R276:N | 3 |  | H227:CD2 | 4.5 |  | E27:OE1 | 2.6 |  | R359:N | 2.9 |
| O4 | G227:N | 3.8 |  |  |  |  |  |  |  |  |  |
| O5 | R276:N | 3.3 |  | H227:CD2 | 3.5 |  | R318:3HD | 2.5 |  | R318:3HD | 3.1 |
| O5 | R276:NH2 | 4.8 |  |  |  |  |  |  |  |  |  |
| O5 | T274:O | 3.8 |  |  |  |  |  |  |  |  |  |
| O6 |  |  |  |  |  |  | A231:1HB | 3.9 |  | S364:OG | 3.8 |
| O6 |  |  |  |  |  |  |  |  |  |  |  |
| **Hydrophobic Contacts** | | |  |  |  |  |  |  |  |  |  |
| C1 | H227:NE2 | 4.2 |  |  |  |  |  |  |  | A231:2HB | 4.3 |
| C2 | H227:NE2 | 4.8 |  | Y281:3HB | 2.6 |  | Q280:NE2 | 4.1 |  |  |  |
| C3 | V23:CG2 | 4.4 |  |  |  |  | L273:CD1 | 3.9 |  | L228:CD2 | 4.2 |
| C4 | E22:2HG | 3.4 |  | M363:3HG | 3.5 |  |  |  |  | L215:CD1 | 3.7 |
| C5 | H227:NE2 | 4 |  | Y281:3HB | 3.3 |  | P272:2HB | 3 |  |  |  |
| C6 | H227:CE1 | 3.9 |  |  |  |  |  |  |  | A231:2HB | 4.3 |
| C7 | H227:3HB | 5 |  | L361:CD1 | 4.4 |  | L361:CD2 | 4.3 |  |  |  |
| C8 | L273:CD2 | 5 |  |  |  |  | P272:2HG | 2.8 |  | L361:CD1 | 5.3 |
| C9 | H227:CG | 3.7 |  |  |  |  | Q280:NE2 | 4.8 |  | R276:NH1 | 4.2 |
| C10 | D224:CA | 4.5 |  | Y281:CZ | 3.7 |  |  |  |  |  |  |
| C12 |  |  |  |  |  |  |  |  |  | H227:NE2 | 3.8 |
| C13 |  |  |  | Y281:OH | 4.2 |  |  |  |  | R359:NH1 | 4.2 |
| C14 |  |  |  | T214:CB | 4.2 |  |  |  |  |  |  |
| C15 |  |  |  |  |  |  |  |  |  |  |  |
| C16 | S275:3HB | 4.5 |  | T214:OG1 | 3.7 |  | V23:O | 5.6 |  | R359:3HB | 3.9 |
| C17 | R276:2HD | 3.8 |  | L215:CD2 | 4.6 |  |  |  |  |  |  |
| C21 |  |  |  |  |  |  |  |  |  | P272:2HG | 4 |
| C22 | H227:CE1 | 4.5 |  |  |  |  | P272:2HG | 3 |  | P358:3HB | 2.8 |
| C23 | G227:C | 5.2 |  |  |  |  | A231:2HB | 5.4 |  |  |  |
| C24 | R276:CZ | 4.2 |  | L228:CD1 | 3.8 |  | S234:3HB | 3 |  | R318:NH1 | 3.3 |
| C25 | L215:CD2 | 4 |  | F270:CE2 | 4 |  | A231:C | 3.9 |  | A231:2HB | 3.8 |
| C26 | L215:CD2 | 3.7 |  | H227:NE2 | 3.8 |  | S364:3HB | 3.3 |  |  |  |
| S1 | D26:3HB | 4 |  | Y281:3HB | 3.6 |  |  |  |  | L228:CD2 | 3.9 |
|  |  |  |  |  |  |  |  |  |  |  |  |

.
